# Influence of the Accelerated Aging Process on the Fragment-Resistant Properties of Para-Aramid Body Armor

**DOI:** 10.3390/ma15186492

**Published:** 2022-09-19

**Authors:** Katarzyna Kośla, Marcin Łandwijt, Michał Miklas, Marzena Fejdyś

**Affiliations:** Institute of Security Technologies “MORATEX”, Marii Sklodowskiej-Curie 3 Street, 90-505 Lodz, Poland

**Keywords:** accelerated ageing, ballistic inserts, para-aramid, fragment resistance, infrared spectroscopy, thermogravimetry, scanning electron microscopy

## Abstract

Para-aramid materials such as Twaron^®^ and Kevlar^®^ are commonly used for ballistic-resistant body armor, which are designed to protect human life and health. For this reason, the materials from which body armor are made should be thoroughly investigated in the area of long-term reliability, particularly with regard to exposure to UV light, humidity and temperature, as these are known causes of degradation in commonly used ballistic materials. This research presents the durability of soft and hard ballistic inserts designed using para-aramid (Twaron^®^) materials. Para-aramid ballistic inserts not subjected to accelerated aging processes and also ones subjected to laboratory aging for 63, 129 and 194 days, which corresponded to 2, 4 and 6 years of aging in real conditions, were tested. The selected para-aramid inserts were verified in terms of ballistic and physico-mechanical properties as well as changes in chemical structure of the ballistic materials. Ballistic tests were carried out with the use of a 1.1 g FSP.22 fragment according to STANAG 2920. Changes in the microstructure of the para-aramid materials were evaluated using infrared spectroscopy and scanning electron microscopy. The obtained results indicate that despite the changes which took place at the molecular level in the Twaron^®^ materials, accelerated aging processes do not affect the fragmentation resistance properties of ballistic inserts made of para-aramid materials.

## 1. Introduction

During exploitation, the materials change their functional properties over time, which is caused by the influence of climatic factors causing gradual deterioration of their molecular structure. This process is called aging. Often, in inferring the time of resistance of materials to weather conditions, it is necessary to carry out aging processes in natural conditions. However, due to the time-consuming nature of these processes, accelerated aging methods are often used, in which intensification of the factors influencing the material accelerates the research process. Accelerated aging studies that predict material behavior can be performed under laboratory conditions in a relatively short time. During this type of research, materials are subjected to controlled changes in parameters, such as temperature, humidity, pressure, intensity of UV radiation or pH, simulating the effects of aging, in order to obtain the necessary data in a limited time [1,2,3,4,5,6].

Aging tests have been performed previously for ballistic materials, such as ultrahigh molecular weight polyethylene (UHMWPE) [7,8,9] or poly(p-phenylene-2,6-benzoxazole) (PBO) [10,11], because they enable verification of the stability of protective parameters and functionality of the inserts, which play a key role in ensuring the protection of users’ life and health.

The influence of aging processes on the ballistic and structural properties of inserts obtained on the basis of aramid materials was the subject of research carried out by Struszczyk et al. [12,13], who showed that the PACVD-modified Style 363/120 woven fabric showed insignificant changes in structural properties after accelerated aging using temperature or simultaneously temperature and humidity as the aging factors. The obtained results confirmed the stability of the PACVD modification during simulated conditions of standard use. In the works of Engelbrecht-Wiggans et al. [5,14], para-aramid yarns from different manufacturers were exposed to various humidity and temperature conditions. The para-aramid materials were found to be resistant to degradation under most aging conditions, showing changes of tensile strength of less than 10% only at 70 °C and 76% relative humidity. The authors determined that the Cunniff and Phoenix–Porwal models accurately determined the changes that were recorded as a result of the experimental work carried out: a decrease in V50 of less than 10%, under the above conditions for approximately 1 year for all studied aramids. Li and co-workers [15] studied the effect of thermal aging on aramid composites obtained on the basis of poly(vinyl butyral)/phenol formaldehyde resin and aramid fabrics with an area density of 410 g/m^2^. In the aforementioned work, the authors showed that the resin consisted of three different phases and exhibited different aging mechanisms that varied with the aging temperature, and they also determined that reduction of the V50 parameter (ballistic limited velocity) depended significantly on the conditions of the aging process.

On the other hand, Konarzewski et al. [16] investigated and evaluated changes of the morphological, physical and mechanical properties of polyaramid, ballistic fibers after natural aging. During the first 2 months of exposition to natural aging, polyaramid fibers showed a color change and a considerable loss in mechanical performance, in particular, an 80% decrease in tensile strength. The results of morphology tests of the fibers after aging showed defibrillation, stress cracking and swelling of polyaramid fibers. Finally, the authors concluded that all samples analyzed after exposure to natural weathering presented hydrolytic degradation and the photochemical effect.

Additionally, Howarter et al. [10] simulated wear-induced damage for single-component fibers of poly(p-phenylene terephthalamide) (PPTA), poly(p-phenylene-benzimidazole-co-p-phenylene terephthalamide) (PBIA-co-PPTA) and PBO. They showed that the mechanical behavior and lifetime of the fibers aged using a custom folding apparatus depends on the chemical and nanostructural composition of fibers. X-ray photoelectron spectroscopy results indicated that the chemical structure of fibers did not change as a result of folding, which suggests that the fibers exhibit primarily mechanical damage and are not affected by abrasion during wear. Moreover, it was determined that although the tensile strength of the fibers was insensitive to molecular scale packing defects detected by Positron Annihilation Lifetime Spectroscopy, the strain to failure decreased as the nanopore content increased.

As a part of this publication, para-aramid materials from the Twaron group were tested. Twaron’s^®^ para-aramids are characterized by the following parameters: density of 1.44–1.45 g/cm^3^, tensile strength from 2.7 to 3.6 GPa, elongation at break which varies from 2.3 to 4.2% and Limiting Oxygen Index (LOI) in the range 29–40% [17,18]. Twaron^®^ is thermally stable, and its decomposition temperature is up to 500 °C. Resistance to organic chemicals is good to excellent as a result of Twaron’s^®^ high crystallinity and strong intermolecular interactions, which prevent chemicals from penetrating the polymer. These properties make it a good choice for body armor, flame-resistant and heat-resistant clothing or ropes and cables. The main problems that arise during the exploitation of para-aramid materials are their quite poor resistance to inorganic chemicals in a low and high pH value. Highly acidic or alkaline chemicals may cause hydrolytic degradation [17,18,19]. Para-aramids are susceptible to UV light, and it is necessary to protect them from exposure to direct sunlight [20].

Twaron^®^ lifetime is determined by the specific combination of load, temperature and other environmental factors. In general, the higher the load and/or the operating temperature, the shorter the lifetime of para-aramid. Additionally, long-term exposure to UV light should be avoided, since this will result in discoloration and negatively influence the mechanical properties of the yarn [21]. The service life for Twaron^®^ ballistic products depends on the way of use and storage. It is estimated that the service life of such products is from 6 to 10 years.

The test results presented in this article were intended to verify the functionality of soft and hard ballistic inserts after simulation of aging processes, and they were then applied to formulate guidelines for the objective determination of time of use and/or storage of the obtained composite systems. Maintaining the stability of the protection and usability parameters of ballistic inserts is an important aspect, especially with regard to their ballistic resistance. In the case of inserts placed on the market, it is possible to subject them to verification tests by comparing the parameters of the ballistic inserts used for a specified period of time with the parameters determined for them when they were new.

Certain complications arise when selling new solutions on the market; therefore, accelerated aging tests are used to determine the time of maintaining their appropriate protective properties. Para-aramid ballistic inserts developed as part of this study should be usable for a period of 6 years from the date of production. Therefore, after carrying out a simulated accelerated aging process corresponding to the period of use in real conditions, the applied ballistic inserts should not show any deterioration of operational properties, including fragment-resistant ones and changes in the physical, mechanical and structural properties of individual components that affect fragment resistance.

In order to confirm the above thesis, an evaluation of the fragment resistance of para-aramid ballistic inserts not subjected and subjected to accelerated aging processes was carried out. For the designed composite solutions, temperature was assumed as the factor-initiating potential structural transformation processes, and in the case of soft ballistic inserts, additionally, fatigue tests involving bending of the para-aramid material were applied. Therefore, to check the properties of ballistic inserts in terms of maintaining their protective parameters during use and/or storage, the results of the tests of resistance to the FSP. 22 (fragment simulating projectile: standardized cylindrical steel projectile with a caliber. 22 designed to emulate the general ballistic performance of irregularly shaped metallic fragments associated with an explosive device) fragment were adopted, as well as the results of structural and physico-mechanical tests performed by infrared spectroscopy, scanning electron microscopy and thermogravimetry.

## 2. Materials and Methods

### 2.1. Materials and Tested Samples

**Figure 1:**
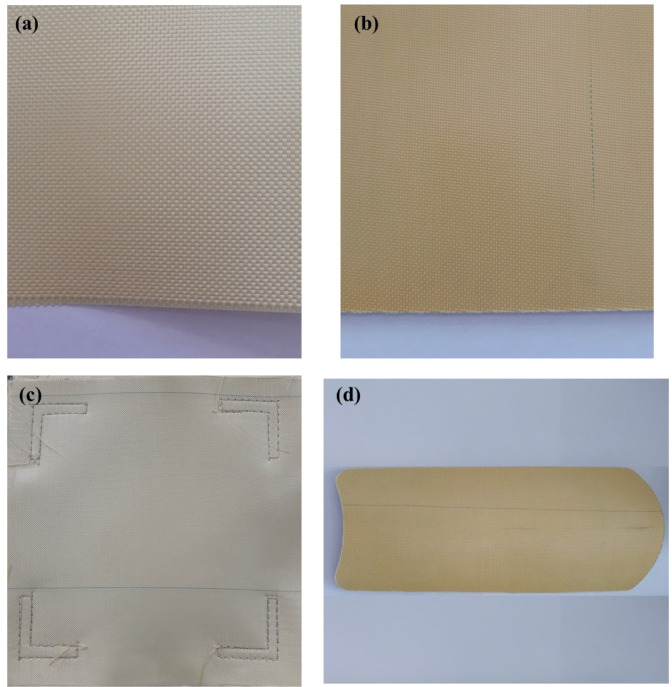
Visual presentation of sample A (**a**), sample B (**b**), ballistic system A (**c**) and ballistic system B (**d**).

Two para-aramid materials were used to obtain the soft and hard ballistic inserts:

- Twaron^®^CT612 WRT (Teijin Aramid GmbH, Wuppertal, Germany), which is plain-woven para-aramid fabric with water repellent treatment (WRT) finishing, which was marked as sample A (Figure 1a). Twaron^®^CT612 WRT has 120 ± 5 g/m^2^ areal density, EPI (ends per inch) of 110 ± 2 per 10 cm, PPI (picks per inch) equal to 110 ± 2 per 10 cm and a linear density of its yarns of 550 and 500 dtex in the warp and weft directions, respectively.
- Twaron^®^CT736 (Teijin Aramid GmbH, Wuppertal, Germany), which is fabric impregnated with polyvinyl butyral (PVB) or PVB phenolic resin (resin amount declared by the manufacturer 12%), which was marked as sample B (Figure 1b). Twaron^®^CT736 fabric is made of 1680 dtex yarns and has a 410 ± 10 g/m^2^ areal density, and the EPI and PPI are equal to 127 ± 4 per 10 cm.
- The objects of the fragment-resistance study were two types of ballistic systems:
- soft ballistic inserts made of Twaron^®^CT612 WRT (Teijin Aramid GmbH, Wuppertal, Germany) para-aramid sheets with 300 × 300 mm dimensions and an areal density of (5.0 ± 0.5) kg/m^2^, which were marked as ballistic system A (Figure 1c);
- hard ballistic inserts obtained from multilayer pressed Twaron^®^CT736 (Teijin Aramid GmbH, Wuppertal, Germany) pre-impregnated sheets with 440 × 160 mm dimensions and an areal density of (10.6 ± 0.5) kg/m^2^, used in conjunction with soft ballistic inserts with the composition and areal density as above, which were marked as ballistic system B (Figure 1d).

The method of preparing soft and hard ballistic inserts has been presented in detail in a previous work [22].

For the physico-mechanical and structural tests, samples of raw materials (Twaron^®^CT612 and Twaron^®^CT736) used in the construction of ballistic inserts with sizes defined in relevant standards and/or test procedures were selected and prepared.

### 2.2. Accelerated Aging Process

The simulation of aging involved incubating the samples in a KMF 240 thermostatic chamber (BINDER GmbH, Tuttlingen, Germany) at a set temperature of 60 ± 2 °C and humidity of 50 ± 2% over a time interval of 63, 129 and 194 days, which corresponded to the time of use of inserts in real conditions of 2, 4 and 6 years, respectively.

The temperature as an ageing factor was selected due to the estimation of environmental factors risk estimation resulting in the conclusion that temperature is most hazardous factor affecting the safety and functionality of the ballistic inserts. The ballistic inserts are protected against UV radiations and humidity by the impregnated textile covers and protected against humidity by WTR. The temperature of 60 ± 2 °C was used because it is below the melting temperature of the para-aramid.

Simulation of the accelerated aging process using the Arrhenius formula, according to ASTM 1980F—2021 ed. “Standard Guide for Accelerated Aging of Sterile Barrier Systems for Medical Devices”, was performed:

AAF = Q_10_^[TAA−TRT/10]^ (1)

where:

AAF: accelerated aging factor;

TAA: accelerated aging temperature [°C];

TRT: storage temperature in the real-time aging of the sample [°C],

Q_10_: aging factor, determined using the kinetics of changes in the selected property/parameter of the material, for temperature changes of 10 °C.

The following equation was used to calculate the actual aging time:

ATT = (365 days)⁄AAF (2)

where:

ATT: the time of accelerated aging, which is equivalent to the real-time aging (corresponding to 2, 4 or 6 years of use under real conditions);

AAF: accelerated aging factor;

The Q_10_ aging factor, which defines the aging curve, was set at the level of 2, and the value of the TRT parameter was assumed to be 25 °C. In turn, the accelerated aging temperature TAA was set at 60 ± 2 °C. According to the guidelines, its value should be within the range from 50 °C to 60 °C, which is related to the fact that a higher temperature significantly reduces the process time; however, it may adversely affect the structural properties of the tested materials, and consequently, the tested materials may show results different from normal.

### 2.3. Fatigue Tests Simulating Mechanical Loads of Ballistic Inserts

The fatigue tests simulating the mechanical loads of ballistic inserts involved bending both ends of the samples in the S 625 device produced by PPU STOGUM (Poland) according to parameters determined on the basis of the following formula [23]:

N = n × x × k (3)

N: the number of deformation cycles on the S 625 device;

N: the number of days of effective insert exploitation per year (values of n were assumed to be equal to 52, 156 or 260);

x: simulated time of use (values from 2 to 6 years were assumed);

k: the number of daily deformations (the value of 30 was taken for investigations).

Therefore, the research defined the number of deformation cycles as:

- 9360 cycles, which corresponded to the estimated use of the insert once a week over 6 years;
- 28,080 cycles, which corresponded to the estimated use of the insert three times a week over 6 years;
- 46,800 cycles, which corresponded to the estimated use of the insert five times a week over 6 years.

The research also assumed a testing angle equal to 30° and a duration of a single cycle of 4 s.

### 2.4. Assessment of Mechanical Properties

The values of the maximum tensile force and elongation at break at maximum force were determined using ISO 13934-1:2013-07 “Textiles—Tensile properties of fabrics—Part 1: Determination of maximum force and elongation at maximum force using the strip method”. The influence of the aging processes on the properties of the aramid composite obtained from multilayer hot-pressed pre-impregnated para-aramid sheets was determined according to ISO 14130: 1997 “Fiber-reinforced plastic composites—Determination of apparent interlaminar shear strength by short-beam method”.

### 2.5. Analysis of Thermal Properties of Para-Aramid Materials using Thermogravimetry (TG)

Thermal analyses were performed using a TGA/DSC3+ thermogravimetric analyzer (Mettler Toledo, Greifensee, Switzerland). The measurement was carried out in an inert gas (nitrogen) atmosphere using an alumina crucible with a capacity of 70 µL. The analysis was performed using the measurement program: heating from 25 °C to 900 °C, heating rate 5 °C/min and/or 10 °C/min. The measurement results are presented in the form of thermograms.

### 2.6. Structural Studies Performed by Infrared Spectroscopy (FT-IR)

An FT-IR analysis was performed on unaged and aged samples, using a NICOLET iS10 spectrophotometer (Thermo Scientific, Waltham, MA, USA) within the wavelength range from 400 to 4000 cm^−1^. Measurements were carried out using the DTGS KBr detector, with a resolution of 4. For FTIR-ATR testing, the material was cut to dimensions of 20 × 20 mm and applied to the crystal by pushing it down in a suitable manner. Both the background spectrum (i.e., the spectrum of the crystal) and the spectrum of the crystal with the sample were measured. The background measurement was saved in the internal memory of the spectrophotometer and automatically subtracted from the sample measurement as a correction for the external conditions of the tests.

### 2.7. Surface Morphology Studies Using Scanning Electron Microscopy (SEM)

Measurements were made using the HR-SEM high-resolution electron microscope (High resolution FEI Nova Nano SEM 450 electron microscope with EDS, Thermo Fisher Scientific Inc., Waltham, MA, USA) with the use of a highly sensitive CBS (Circular Backscatter) detector with 4 concentrically arranged sectors, enabling the detection of backscattered electrons (BSE) and cooperating with the electron energy deceleration mode. The measurements were carried out at accelerating voltage values of 3 and 5 kV and under magnifications of 500× and 8000×.

### 2.8. Fragment Resistance Measurements

Ballistic limited velocity (V50) was measured at ambient temperature according to STANAG 2920 “Ballistic test method for personal armour materials and combat clothing”. The V50 is the average of the velocities recorded for six fragment impacts consisting of the three lowest velocities for complete penetration and the three highest velocities for partial penetration, provided that the spread is not greater than 40 m/s. The FSP.22 standard fragment with a mass of 1.10 ± 0.03 g, diameter 5.38 ± 0.02 mm and length 6.35 mm, made of steel with a hardness of 30 ± 2 HRC was used for the fragment resistance tests. The research was carried out in accordance with the methodology presented in the previous work [22].

## 3. Results

### 3.1. Results of Structural, Physico-Mechanical and Thermogravimetric Tests of Para-Aramid Materials Subjected to Accelerated Aging Processes

**Figure 2:**
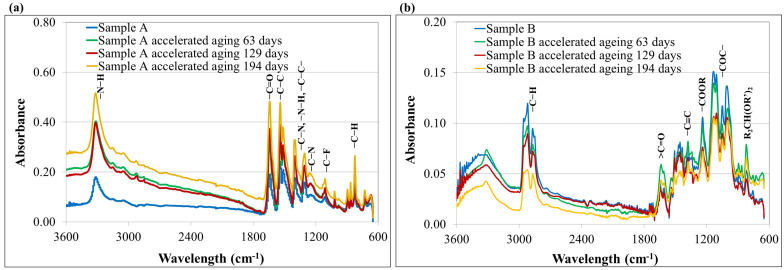
FT-IR spectra of sample A (**a**) and sample B (**b**) non-aged and subjected to accelerated aging processes.

The spectra obtained by infrared spectroscopy are presented in Figure 2.

The spectra (Figure 2a) obtained for sample A (before and after accelerated aging process) were characterized by bands with wavenumber values of 725–951 cm^−1^, corresponding to the bending vibrations of C−C and C−H bonds in an aromatic ring. Bands of low intensity and wavelength values equal to 1012 cm^−1^ and 1132 cm^−1^ probably corresponded to the C−F bond present in fluoroacrylic compounds, which are the part of the hydrophobic layer of the para-aramid material. The presence of a band occurring in the wavenumber range of 1250 cm^−1^, corresponding to the bending vibrations of C−N and/or P−O bonds derived from para-aramid compounds and/or antistatic agents and lubricants of WRT finishing, was also indicated. In addition, the presence of bands with the wave numbers of 1306 cm^−1^ and 1397 cm^−1^ was observed, coming from the stretching vibrations of the C−N, N−H and C−C bands. Based on the analysis of the obtained FT-IR spectra, the presence of bands characteristic for vibrations of the C=O groups could be noted, at wavelengths equal to 1636–1643 cm^−1^. The bands in the wavenumber of 3315 cm^−1^, characteristic of the stretching vibrations of N−H bonds, indicate the presence of amine groups in the molecule. The FT-IR spectra also showed bands in the range 1511–1540 cm^−1^ from the stretching vibrations of C−C bonds occurring in the aromatic ring. The obtained FT-IR spectra corresponds well with literature reports for Twaron^®^ para-aramides [12,24].

The spectra obtained for the aged samples A showed an increase in the intensity of the bands, especially in the range of 1397–1643 cm^−1^ characteristic for the vibrations of the C=O groups as well as N−H, C-N and C−C, which is consistent with the literature data [25,26,27] and may indicate the degradation of 1,3-dihydroxyalkyl- 5,5-dialkylhydantoin contained in the WRT finishing and/or para-aramid fibers in the presence of humidity and atmospheric oxygen, caused by the cleavage of amide bonds.

The FT-IR spectrum of sample B is shown in Figure 2b. The FT-IR spectra contained a characteristic peak for the −OH group, shown at 3322 cm^−1^, which was a little broader, deviating from its normal value of ~3600 cm^−1^, to show the presence of intermolecular hydrogen bonding. The presence of a band occurring in the wavenumber range of 2870–2954 cm^−1^, corresponding to the vibrations of C−H bonds in −CH_3_, −CH_2_ and −CH aliphatic groups, was also indicated. The peak for the carbonyl group appeared at 1709 cm^−1^ [28]. The FT-IR spectra also showed bands in the range of 1473–1341 cm^−1^ from the vibrations of bonds between carbon atoms occurring in the alkynes. The band in the wavenumber of 1243 cm^−1^, characteristic for the vibrations of −COOR bonds, indicated the presence of ester groups in the molecule. Based on the analysis of the obtained FT-IR spectra, the presence of bands characteristic for vibrations of the C−O−C groups could be noted, at wavelengths equal to 1105–1134 cm^−1^, as well as for the acetal group at the 996 cm^−1^ wavelength [29]. The obtained spectrum indicated the presence of a polyvinyl butyral (PVB) resin in sample B.

After the accelerated aging process, we observed a decrease in the intensity of the vibration bands of practically all functional groups in the molecules of the tested material, except for the carbonyl group > C=O, which occurred at the wave number value of 1646 cm^−1^, which may indicate changes taking place at the molecular level in the PVB resin resulting from the thermo-oxidation process and leading to the formation of α, β-unsaturated carbonyls and/or dienes [30]. Additionally, a shift of the band presented at 3322 cm^−1^ towards the lower wavenumber values of 3295 cm^−1^ and a change of its character from broad to sharp could be observed, which may indicate the formation of the band which is characteristic of N-H bonds in para-aramid molecules.

**Figure 3:**
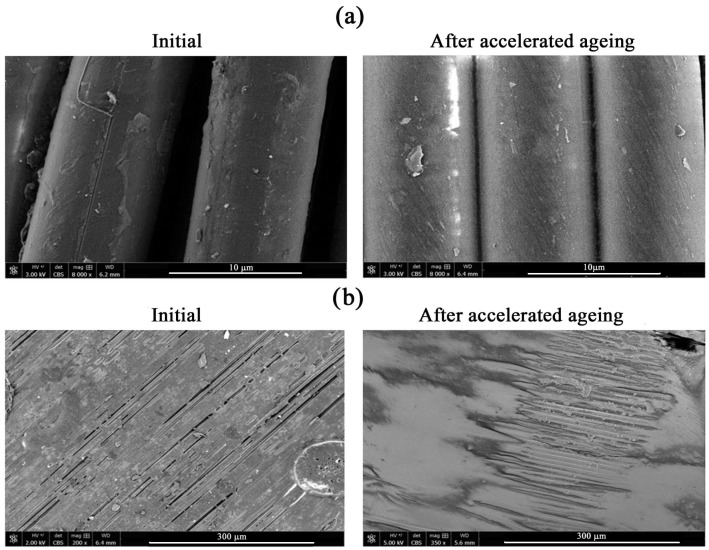
SEM images obtained for sample A (**a**) and sample B (**b**) before and after accelerated aging processes.

In addition, scanning electron microscopy (SEM) tests were performed, the results of which are presented in Figure 3. The acquisition of SEM images was aimed at verifying possible changes in the morphology of para-aramid materials under the influence of accelerated aging processes.

Structural changes were observed in the tested sample A of para-aramid material on the SEM images, under a magnification 8000×. The images show that the fibers of sample A subjected to the accelerated aging processes became smoother and more homogeneous, and the lumps and roughness appearing on their surface were much smaller than in the case of non-aged para-aramid fibers. These changes may confirm the results obtained during FT-IR and TG analyses and indicate oxidation of the components of the WRT finishing.

SEM images also enabled observation of the resin structure on the surface of fibres from sample B, which enables production of the composite in the process of thermal-pressure pressing. The film layer was not continuous, and there were free spaces in it where aramid fibers and heterogeneity of the structure were visible. Due to the heterogeneity and the presence of numerous defects, it was unfortunately not possible to determine the effect of accelerated aging processes on changes in the morphology of sample B.

**Figure 4:**
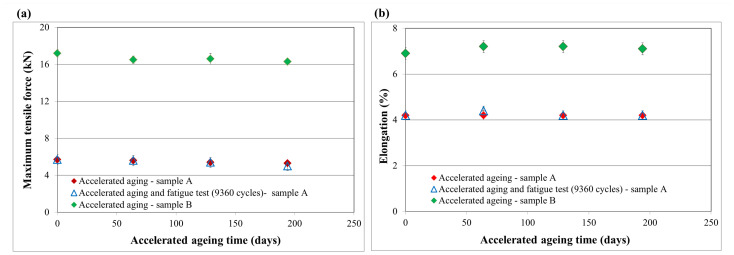
Maximum tensile force (**a**) and elongation (**b**) results for samples A and B.

The results of the maximum tensile force and elongation obtained for the sample A and B used in the construction of ballistic inserts subjected to the accelerated aging process are presented in Figure 4.

The results obtained for sample A (Figure 4a) showed a slight decrease in the value of the maximum tensile force (towards the matrix) for the tested samples as the time of accelerated aging increased. The highest reduction of the maximum tensile force, amounting to 7%, was recorded for samples subjected to accelerated aging processes for the period of 194 days, which corresponded to the period of 6 years of insert usage. A similar dependence (over 12% reduction in the value of the maximum tensile force) was also observed for samples subjected to the fatigue test involving bending of the ballistic inserts in the amount of 9360 cycles (this value corresponded to the use of the ballistic insert once a week for 6 years) and then aging processes corresponding to a period of 6 years of use. The results obtained for sample B (Figure 4a) also showed a slight decrease in the value of the maximum tensile force for the tested samples as the time of accelerated aging increased. The 5% reduction of the maximum tensile force was recorded for samples subjected to accelerated aging processes, which corresponded to the period of 6 years of insert usage. In none of the tested variants were there any changes in the value of the relative elongation parameter at the maximum force for para-aramid material exceeding the measurement error (Figure 4b).

In turn, the composite obtained by thermal-pressure pressing of the Twaron^®^CT736 para-aramid material, sample B, was tested for interlaminar shear strength. For the sample of the composite not subjected to the accelerated aging processes, the value of interlaminar shear strength was 2.11 ± 0.27 MPa, while the composite after the accelerated aging process showed the value of 1.30 ± 0.39 MPa. Therefore, the sample B subjected to accelerated aging processes, corresponding to a period of 6 years of use, showed over 38% lower bending elongation values in the interlaminar shear strength tests carried out than an unaged composite.

**Figure 5:**
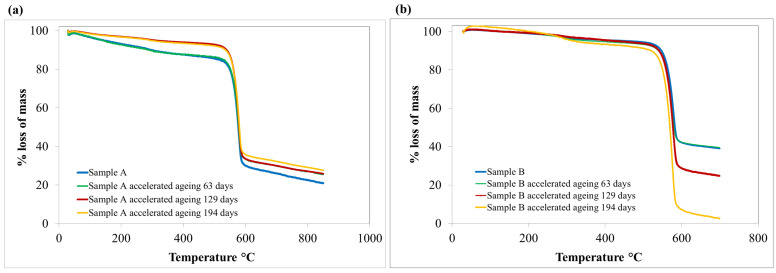
Thermogravimetric curves of sample A (**a**) and sample B (**b**).

The samples A and B, non-aged and subjected to accelerated aging process, were also characterized in terms of substance mass change, depending on temperature changes, using thermogravimetric analysis. The results are presented in Table 1 and Figure 5.

The results of the TG tests indicated a significant decrease in the temperature values for which there was a 5% mass loss of sample A and sample B of para-aramid materials as a result of accelerated aging processes, especially in those simulating the period of 4 and 6 years of ballistic insert use. In the case of sample A, after the accelerated aging process corresponding to the period of 6 years of use, the T5 temperature was reduced by 17% (from 355 °C to 294 °C), and for sample B, it was reduced by even 31%.

The decrease in the temperature value at which there was a 5% loss in the mass of para-aramids may indicate changes taking place at the structural level after the aging processes. It is assumed that, in the case of sample B, the changes in the obtained value of the T5 parameter were related to structural changes of the PVB resin present in the pre-impregnate. Upon exposure to atmospheric air, this resin can slowly oxidize, leading to the formation of unsaturated carbonyl compounds, which are likely to have lower decomposition temperatures. The longer the aging process, the more resin is oxidized and the lower the T5 temperature values that can be obtained. On the other hand, sample A is a material with a WRT finish, which gives a hydrophobic character to the para-aramid fabric. The WRT finishing is a mixture of fluoropolymers such as fluoroacrylate polymers, antistatic agents (e.g., alkylphosphate compounds) and lubricants that reduce the resistance of machine elements used in production processes (e.g., a mixture of 1,3-dihydroxyalkyl-5,5-dialkylhydantoin, oleic acid ester and ethylene oxide) [31]. A decrease of the T5 value with increasing aging time may be related to the transformation/degradation of compounds included in the WRT finish of the aramid fabric and/or oxidation of the surface of the para-aramid during the aging processes.

On the other hand, the temperature values for 50% mass loss (T50) for both samples A and B ranged from 569–583 °C, which is consistent with the available literature data [32] and practically did not change (temperature change below 2.5%) after the accelerated aging process compared to the reference sample. The T50 temperatures corresponding to the decomposition of the para-aramid assumed high values, which did not change for both tested materials after the accelerated aging processes. This means that the para-aramid showed significant thermal stability [33].

The above theorem was confirmed by the results of the TG analysis carried out for the raw para-aramid material (without WRT finishing or resin) (Supplementary Materials) [18]. These results indicate that before and after aging simulating 6 years of usage life of raw material, the T5 values were slightly above 540 °C, while the T50 values were between 587–588 °C. The T50 temperatures of the raw para-aramid were very similar to those obtained for samples A and B. On the other hand, the T5 values of raw material were much higher (increase of the T5 parameter from 85 °C to even 294 °C) in comparison with T5 of samples A and B.

Based on the obtained results of thermal stability tests, we conclude that the stability determined by the T5 index for samples A and B is responsible for information related to changes in the finish (WRT or PVB resin) of para-aramid fabrics. The T50 index indicates the thermal stability of the para-aramid material.

Nevertheless, the differences in the intensity of the bands in the FT-IR spectrum, indicating changes at the molecular level of the resin existing in sample B, occurring during the accelerated aging processes, and the differences in the inter-laminar shear strength practically do not affect the ballistic properties of the para-aramid composite, i.e., resistance to the FSP.22 fragment, as presented in Section 3.2 of this article.

### 3.2. Fragment Resistance of Para-Aramid Ballistic Inserts Subjected to Accelerated Aging and Fatigue Tests

**Figure 6:**
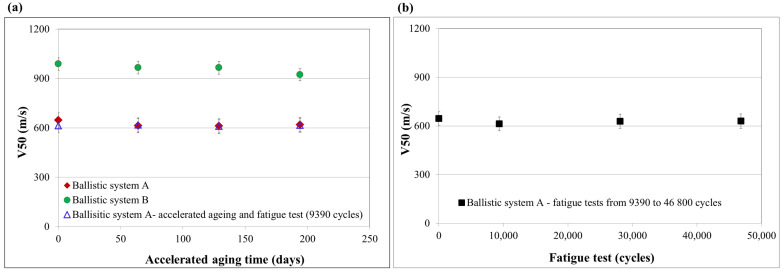
Fragment resistance of para-aramid inserts after accelerated aging cycles (**a**) and fatigue tests (**b**).

The samples of ballistic composites intended for the assessment of resistance to debris were soft ballistic inserts made of Twaron^®^CT612 (ballistic system A) and hard ballistic inserts obtained by thermal-pressure pressing of Twaron^®^CT736 sheets combined with the above-mentioned soft ballistic inserts (ballistic system B). The samples were tested for the determination of fragment resistance (V50 parameter) after cycles of accelerated aging simulating the usage life of ballistic composites ranging from 2 to 6 years. The test results (Figure 6a) were summarized and compared with the data obtained for composites not subjected to accelerated aging cycles.

The tested composites, which made up the soft ballistic inserts (ballistic system A) and hard ballistic panels (ballistic system B), practically did not show any changes in resistance to the FSP.22 fragment after the accelerated aging processes, simulating 2, 4 and 6 years of use of the composites (changes in the range of up to 6% were noted, falling within the measuring error).

Moreover, fragment resistance tests were performed for ballistic systems A after:

- Fatigue that simulated the use of inserts one, three and five times a week over a period of 6 years (Figure 6b),
- Fatigue simulating the use of the insert once a week (9360 fatigue cycles) and additionally accelerated aging in the period corresponding to 2–6 years of use (exposure to the temperature of 60 °C for a period of 63 to 194 days) (Figure 6a).

Ballistic system A showed only changes within the measurement error (~5%) of the V50 parameter for the FSP.22 fragment after the fatigue tests, with both fatigue tests corresponding to the use of the insert once a week and in the accelerated aging processes simulating 2 to 6 years of use of the inserts. Therefore, it should be considered that the developed ballistic system A does not lose its functional properties as a result of fatigue tests and accelerated aging processes simulating the 6-year usage life of the inserts.

The research results presented above for para-aramid ballistic systems are similar to investigations performed in the field of accelerated ageing’s impact on properties of UHMWPE (Dyneema^®^) [7,8]. Chabbas et al. [7] showed the effect of accelerated aging at 65 °C and 80% relative humidity, which corresponded to 5 years of usage at 35 °C on the V50 parameter of Dyneema^®^ SB21, SB31 and SB61 composites. The results indicated that Dyneema^®^ materials retained their ballistic (V50) performance and exhibited a good lifetime expectancy. In turn, Fejdyś and co-workers [8] presented the impact of the ageing factors, progressing with the time of exposure, which resulted in the deterioration of mechanical properties and changes in the structural properties of Dyneema^®^ SB51 and Dyneema^®^ HB26 sheets. Minor changes in the structure of the composite occurred only for samples subjected to ageing for 150 days (about 6.5 years of usage). These changes were primarily related to the reactions of breaking, oxidation and crosslinking processes of polymer chains. The studies also showed that in the case of soft ballistic inserts subjected to accelerated ageing, there was no significant difference between the values of the mean substrate deflection as compared to the results obtained for ballistic inserts which had not been subjected to conditioning cycles. As in the case of Dyneema^®^, changes in structural and some physico-mechanical properties were achieved for para-aramid materials as a result of the aging processes of the material, but the ballistic, protective abilities of inserts were maintained.

## 4. Conclusions

Based on the obtained test results, it was determined that the investigated para-aramids had undergone post-processing, as a result of which they were covered with a layer of hydrophobic WRT finish (sample A) or PVB resin (sample B). The fibers from which the Twaron^®^CT612 para-aramid material is made, used to make soft ballistic inserts, slightly change their structural properties, due to the degradation of the WRT finishing of para-aramid in the presence of oxygen, moisture and elevated temperature, which results in lower temperatures for 5% loss of mass and reduction of the maximum tensile force obtained for this material. Soft ballistic inserts (ballistic system A) do not change their fragment-resistant properties under the influence of accelerated aging processes, corresponding to their 2-, 4- and 6-year service life and as a result of fatigue tests simulating their use at a frequency of 1, 3 or 5 times a week over a period of 6 years.

The ballistic system B subjected to aging processes showed a reduction of the maximum tensile force and over 38% reduction in the value of the interlaminar shear strength. In addition, for the para-aramid material from which the above-mentioned composite was made, we also observed a decrease in the intensity of the vibration bands of practically all functional groups found in the molecules of the tested material, except for the carbonyl group > C=O, which may indicate changes taking place at the molecular level in resin covering the composite, taking place during the accelerated aging processes.

Despite the changes taking place at the molecular level in sample A and sample B, soft and hard inserts (ballistic system A and B) made of para-aramid materials do not deteriorate in terms of their resistance to FSP.22 fragments under the influence of accelerated aging processes corresponding to 2-, 4- and 6-year periods of their use. Such inserts can be used for a period of at least 6 years from the date of their production.

In view of the above, as a result of the aging process, the degradation processes of the hydrophobic finish and resin take place, and the para-aramid fabrics themselves will not significantly change their properties, as have been evidenced by the lack of ballistic properties changes of body armor.

## Figures and Tables

**Table 1:** The 5% and 50% temperature-induced mass loss of sample A and sample B para-aramids.

No.	Tested Sample	Parameter	Before Ageing	Accelerated Ageing 63 Days	Accelerated Ageing 129 Days	Accelerated Ageing 194 Days
1	Sample A	T5 [°C]	355	351	309	294
2		T50 [°C]	576	578	580	580
3	Sample B	T5 [°C]	459	425	367	317
4		T50 [°C]	583	576	581	569

## Data Availability

Not applicable.

## Supplementary Materials

The following supporting information can be downloaded at: https://www.mdpi.com/article/10.3390/ma15186492/s1, Table S1: 5% and 50% temperature-induced mass loss of the raw para-aramid material; Figure S1: Thermogravimetric curves of the raw para-aramid material; Table S2: 5% and 50% temperature-induced mass loss of sample A and B (TG analysis performed in air atmosphere); Figure S2: Thermogravimetric curves of sample A and B obtained in air atmosphere.
