# Supplementary material for: Influence of the Accelerated Aging Process on the Fragment-Resistant Properties of Para-Aramid Body Armor
Source: Materials (Basel). 2022 Sep 19;15(18):6492. doi: 10.3390/ma15186492 (PMC9502209; doi:10.3390/ma15186492)

Supplementary materials

Influence of accelerated aging process on the fragment-resistant properties of para-aramid body armor

Table S1. 5% and 50% temperature-induced mass loss of the raw para-aramid material.

No.	Tested sample	Parameter	Before ageing	Accelerated ageing 194 days
1	Raw	T5 [°C]	544	542
2		T50 [°C]	587	588

Figure S1. Thermogravimetric curves of the raw para-aramid material.

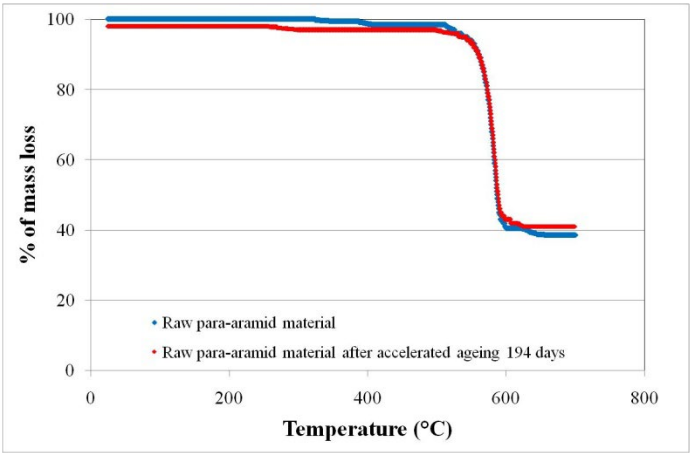

Table S2. 5% and 50% temperature-induced mass loss of sample A and B (TG analysis performed in air atmosphere).

No.	Tested sample	Parameter	Before ageing	Accelerated ageing 194 days
3	Sample A	T5 [°C]	310	406
4		T50 [°C]	547	548
5	Sample B	T5 [°C]	304	452
6		T50 [°C]	548	551

Figure S2. Thermogravimetric curves of sample A and B obtained in air atmosphere.

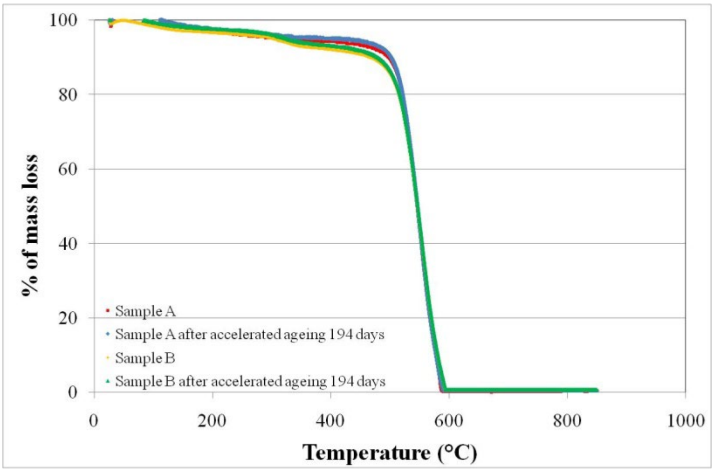

Supplement: Supplementary file 1 [file materials-15-06492-s001.zip › materials-1899648-supplementary.pdf]
